# EHMTI-0092. Activation of the 5-HT2B receptor induces dural plasma protein extravasation in a mouse migraine model

**DOI:** 10.1186/1129-2377-15-S1-F11

**Published:** 2014-09-18

**Authors:** A Hunfeld, M Kremser, D Segelcke, H Lübbert

**Affiliations:** 1 Animal Physiology, Faculty of Biology and Biotechnology, Bochum, Germany

## Introduction

Migraine attacks originate in the meninges which are densely innervated by trigeminal nerve fibers. Upon stimulation, endothelial cells of dural blood vessels secrete nitric oxide and as a consequence, neuropeptides are released from trigeminal nerve fibers. This, in turn, leads to meningeal plasmaproteinextravasation (PPE) and vasodilation. Inflammatory components such as PPE and vasodilation serve as indicators for migraine attacks in animal models. A guinea pig migraine model was described in which the partial 5-HT2B/2C receptor agonist meta-Chlorphenylpiperazine (mCPP) is applied to induce PPE in the dura mater. We have transferred this model to mice.

## Aim

Characterize the role of the 5-HT2B in a mouse migraine model.

## Methods

Tissue accumulation of the tracer Evans Blue is measured to quantify the extent of the PPE.

## Results

Mice are only responsive to mCPP after several weeks of hypoxia. The induction of PPE in hypoxic mice is blocked by the specific 5-HT2B receptor antagonist BF-1.

## Conclusion

The 5-HT2B receptors play a crucial role in the induction of migraine symptoms in hypoxic mice.

No conflict of interest.

